# Loperamide Therapy for Acute Diarrhea in Children: Systematic Review and Meta-Analysis

**DOI:** 10.1371/journal.pmed.0040098

**Published:** 2007-03-27

**Authors:** Su-Ting T Li, David C Grossman, Peter Cummings

**Affiliations:** 1 Department of Pediatrics, University of California Davis, Sacramento, California, United States of America; 2 Department of Preventive Care, Group Health Cooperative, Seattle, Washington, United States of America; 3 Center for Health Studies, Group Health Cooperative, Seattle, Washington, United States of America; 4 Department of Health Services, University of Washington, Seattle, Washington, United States of America; 5 Department of Pediatrics, University of Washington, Seattle, Washington, United States of America; 6 Department of Epidemiology, University of Washington, Seattle, Washington, United States of America; Royal West Sussex National Health Service Trust, United Kingdom

## Abstract

**Background:**

Loperamide is widely used in adults for acute diarrhea. However, its use in children has been discouraged by the World Health Organization and the American Academy of Pediatrics owing to concerns over safety and efficacy in young children.

**Methods and Findings:**

To assess the efficacy and adverse effects of loperamide compared with placebo for acute diarrhea in children, we reviewed Medline, EMBase, the Cochrane Central Register of Controlled Trials, and bibliographies of known clinical trials and of review articles, and we also interviewed key investigators in the field. We undertook a systematic review and meta-analysis of randomized controlled trials of children younger than 12 y of age with acute diarrhea, comparing loperamide with placebo. Included trials reported data on diarrhea duration or severity, or provided data on adverse effects. Compared with patients who received placebo, patients allocated to loperamide were less likely to continue to have diarrhea at 24 h (prevalence ratio 0.66, 95% confidence interval [CI]: 0.57 to 0.78), had a shorter duration of diarrhea by 0.8 d (95% CI: 0.7 to 0.9 d), and had a lower count of stools at 24 h (0.84, 95% CI: 0.77 to 0.92). Results were similar when random-effects summaries were estimated. Serious adverse events, defined as ileus, lethargy, or death, were reported in eight out of 927 children allocated to loperamide (0.9%, 95% CI: 0.4% to 1.7%). Serious adverse events were not reported in any of the 764 children allocated to placebo (0%, 95% CI: 0% to 0.5%). Among the children allocated to loperamide, serious adverse events were reported only among children younger than 3 y.

**Conclusions:**

In children who are younger than 3 y, malnourished, moderately or severely dehydrated, systemically ill, or have bloody diarrhea, adverse events outweigh benefits even at doses ≤0.25 mg/kg/d. In children who are older than 3 y with no/minimal dehydration, loperamide may be a useful adjunct to oral rehydration and early refeeding.

## Abstract 2

In seriously ill children under 3 years of age with diarrhea the adverse effects of loperamide outweighed the benefits, but the drug could be useful as part of treatment for other children.

## Editors' Summary

**Background.:**

While diarrhea is often thought of as a mild, inconvenient condition, it is estimated that, worldwide, 1.6–2.5 million children under 5 y old die each year from diarrhea, most of them in developing countries. Dehydration is the key factor in the deaths of these children. In richer countries, diarrhea is rarely deadly, but it has been calculated that, in the United States, the annual national health-care cost associated with the condition amounts to around US$1.5 billion. Some of the cost results from the purchase of anti-diarrheal drugs. Loperamide is one of the most widely used of these drugs. In most countries, it can be obtained without a prescription. The use of loperamide is intended to reduce the frequency of bowel movements, but taking it will not lead to rehydration, nor will it kill the infectious organisms responsible for the condition.

**Why Was This Study Done?:**

The World Health Organization and other health authorities have concerns that loperamide may not be effective in young children and that it may not be safe. In the United States, the Food and Drug Administration approves its use for children older than 2 y of age. The researchers wanted to know whether loperamide could play a useful part in treating diarrhea in children.

**What Did the Researchers Do and Find?:**

They did not do any new work with children suffering from diarrhea. Instead, they searched the medical literature for previously conducted trials involving the use of loperamide. They used these previously conducted trials to estimate whether use of loperamide influenced the duration of diarrhea or the number of diarrheal stools in children under 12 y of age. They also used these trials to examine adverse effects of loperamide. In total they found 13 studies that met the criteria they had set for inclusion in their study. More than 900 children in these studies had been given loperamide for their diarrhea; each trial also had a control group of children whose treatment did not include loperamide. Most of the children in the studies had only mild diarrhea. The researchers found that, compared with children in the control groups, those treated with loperamide were less likely to continue to have diarrhea 24 h later, had a shorter duration of diarrhea, and had a lower count of diarrheal stools. However, eight of the children given loperamide and none of the control group did have serious adverse effects. All those who had serious adverse effects were less than 3 y of age.

**What Do These Findings Mean?:**

The researchers concluded that if a child is less than 3 y of age, malnourished, moderately or severely dehydrated, or has bloody diarrhea, the risk of adverse events from loperamide treatment outweighs the benefits, even at low doses. In other children, loperamide may be a useful part of treatment. However, they advise that rehydrating the child (by giving fluids orally) and progressively returning him or her to a normal diet should still be the main focus in the treatment of childhood diarrhea.

**Additional Information.:**

Please access these Web sites via the online version of this summary at http://dx.doi.org/10.1371/journal.pmed.0040098.

- For advice on the treatment of diarrhea, visit the Web sites of BestTreatments (produced by BMJ Publications) and of FamilyDoctor (produced by the American Academy of Family Physicians)
- The World Health Organization has a Web page about diarrhea that gives a global perspective on this major cause of childhood death
- UNICEF (the children's organization of the United Nations) includes diarrhea in its *Facts for Life* series, which aims to provide parents and other caregivers with the information they need to save and improve children's lives; the messages contained in *Facts for Life* are based on the latest scientific findings, but are presented in nontechnical language

## Introduction

Worldwide, children younger than 5 y of age have approximately three cases of diarrhea annually and 1.6–2.5 million children younger than 5 y die each year from diarrhea, as estimated from studies published between 1992 and 2000 [1]. The national health-care cost for diarrhea-associated disease in the United States was estimated to be US$1.55 billion in 2002 [2]. In a 1997 survey, an estimated 34% of persons with a diarrheal illness in the United States reported taking anti-diarrheal medications [3]. Some review papers on the treatment of acute diarrhea in adults suggest loperamide as a first-line agent [4,5]. Although loperamide is widely used in adults, the World Health Organization and the American Academy of Pediatrics are concerned about its use in young children because of concerns over its efficacy and safety [6,7]. In the United States, loperamide is approved by the Food and Drug Administration for use in children older than 2 y of age.

We conducted a systematic review and meta-analysis of randomized controlled trials to estimate whether loperamide use influenced the duration of diarrhea or the number of diarrheal stools in children younger than 12 y of age with acute diarrhea (see Text S1 for the QUOROM checklist). We also searched the literature for information about adverse effects of loperamide.

## Methods

### Data Sources

First, we searched the following databases: Medline (1966–2006; http://www.ncbi.nlm.nih.gov/entrez/query.fcgi?DB=pubmed), EMBase (1988–2006; http://www.embase.com), and the Cochrane Central Register of Controlled Trials (http://www.mrw.interscience.wiley.com/cochrane/cochrane_clcentral_articles_fs.html, with the search carried out on 24 April 2006) using the search terms “diarrhea/”, “diarr$(tw)”, “diarrhea(tw)”, and “loperamide/” or “imodium” [8]. In the Medline and EMBase databases, randomized studies were identified by limiting our studies to “randomized controlled trial”, “multicenter study”, “controlled clinical trial”, or “clinical trial”. Second, we searched bibliographies of known clinical trials and of review articles for other eligible publications. Third, we contacted major health organizations that focus on diarrhea prevention and treatment, key investigators in the field, and major pharmaceutical companies that manufacture loperamide (McNeil Consumer Healthcare, http://www.imodium.com) to ask for details of other known clinical trials. Primary authors were contacted to obtain additional information if necessary. Conference proceedings were not searched. Publications were included in all languages.

### Study Selection

We evaluated each trial for inclusion in the review on the basis of four criteria: study design (randomized controlled trial), study population (children younger than 12 y of age with acute diarrhea), intervention (loperamide versus control), and availability of outcome data on diarrhea duration or severity. Studies fulfilling all four criteria were included. Studies of the combination of loperamide and another drug, such as an antibiotic, were not included.

### Outcome Measures

Our primary outcomes of interest were the characteristics of the clinical course of diarrhea and the incidence of adverse events. Measures of diarrhea intensity included duration (in days), frequency (number of stools per day), and stool volume. Serious adverse events were considered to be ileus, lethargy, or death.

### Methodological Quality of Studies

We recorded four aspects of study design: allocation concealment, generation of allocation sequence, blinding, and inclusion of all randomized participants. Allocation concealment, generation of allocation sequence, and blinding of the treatment assessor were categorized as adequate, not adequate, or unclear. Allocation concealment was categorized as adequate (patients and investigators enrolling patients cannot foresee assignment), inadequate (authors did not report an allocation-concealment approach or reported an approach that could not be considered adequate), or unclear (allocation concealment was stated, but the method used was not described). Generation of allocation sequence was categorized as adequate (sequences are suitable to prevent selection bias, and method used is described), inadequate (sequences could be related to prognosis), or unclear (generation of allocation sequence was stated, but method was not described). Blinding was categorized as double-blind (neither patient nor care provider/assessor knows which treatment is given), single-blind (patient or care provider/assessor is aware of treatment given, and open (all parties are aware of treatment). Inclusion of all randomized participants was categorized as adequate if more than 90% of randomized patients were included in the intention-to-treat analysis, or inadequate if it was unclear or if fewer than 90% of patients were included in the intention-to-treat analysis. Studies were not excluded on the basis of methodological quality of trials, but this information was used in the sensitivity analysis.

### Data Abstraction

Data were extracted independently by two authors (STL and DCG) and any disagreements were resolved by consensus.

### Study Characteristics

Descriptive data for each trial included details of participants (i.e., age), country of trial, stool pathogens, details of intervention (including study medication and dose), and definition of outcomes. Our assessment of clinical heterogeneity was focused on loperamide dose and definition of diarrhea resolution across the trials.

### Statistical Methods

Publications with common outcome measures were included in a meta-analysis. We summarized risk ratios and mean differences across studies primarily using the fixed-effects method: the Mantel-Haenszel method for dichotomous outcomes, or the inverse variance method for continuous outcomes [9–11]. Data on diarrhea counts in the first 24 h were summarized using the log of the count ratio from each study and inverse variance weights [12,13]. We used the random-effects method of DerSimonian and Laird to see if this changed the results [9–11]. We performed tests for heterogeneity using the Mantel-Haenszel or inverse variance methods [9–11].

Adverse outcomes were rare; to estimate the 95% confidence intervals (CIs) for the cumulative risk of an adverse event in each trial arm, we used exact (Clopper-Pearson) binomial methods [9,14,15]. To obtain pooled risk difference estimates, we used the Mantel-Haenszel method [9–11].

Subanalyses were planned a priori to determine whether dose of loperamide, definition of diarrhea resolution, location of subject population (outpatient or inpatient), methodological quality, or infectious agent (bacteria or virus isolated from stools) modified the effect of loperamide on diarrhea or explained any heterogeneity seen. Preplanned subanalyses were performed only if the characteristic of interest was reported in three or more studies.

All analyses were performed using Stata 8.0 (Stata, College Station, Texas, United States) [9,10,12].

## Results

### Study Selection

**Figure 1:**
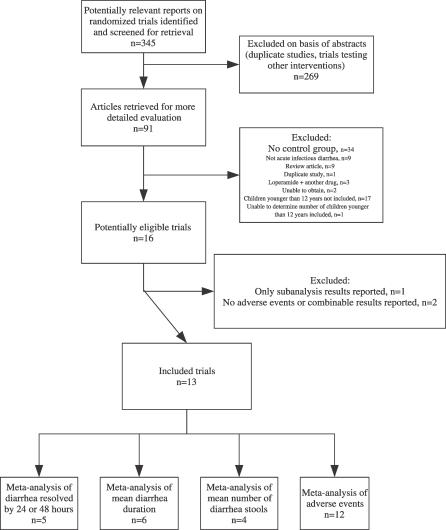
Summary of Meta-Analysis Flow Some studies reported more than one outcome that could be summarized.

The initial search for studies involving loperamide treatment of infectious diarrhea yielded 345 articles. Review of the abstracts and exclusion of irrelevant and duplicate articles yielded 91 articles (Figure 1). Key researchers in the field did not suggest any additional trials. Of the 91 articles examined, we excluded 34 studies with no control group, 16 studies where children younger than 12 y of age were not included, one study where we were unable to determine the number of children younger than 12 y of age, and 24 studies that did not otherwise meet inclusion criteria, leaving 16 studies that met the four inclusion criteria (Figure 1) [16–31]. We excluded one study because only subanalysis results were presented [29]. An additional two studies were not included in the systematic review because neither adverse effects nor outcome data that could be combined were available in either of the studies [28,30].

### Study Characteristics

The 13 included trials studied 1,788 children younger than 12 y: 975 children assigned to loperamide and 813 assigned to placebo. Of the 13 studies, six studies used loperamide doses ≤0.25 mg/kg/d, four studies used doses >0.25 mg/kg/d, and three studies used unclear maximum doses (Table 1).

**Table 1:**
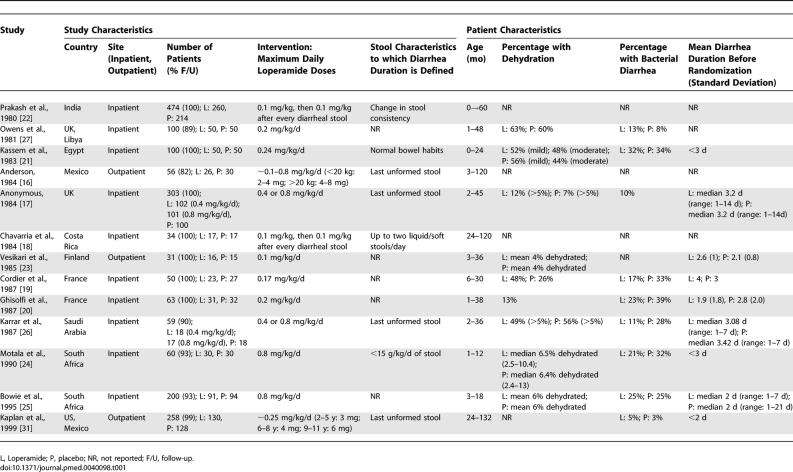
Characteristics of the Studies Included in the Meta-Analysis of Use of Loperamide for Acute Diarrhea

Definitions of diarrhea resolution were not uniform across studies (Table 1). While four out of 13 studies defined diarrhea resolution as the time to the last unformed stool [16,17,26,31], four studies defined diarrhea resolution as change in stool consistency [22], <15 g of stool/kg/d [24], two or fewer liquid/soft stools/d [18], or return to normal bowel habits [21], and five studies did not report their criteria for diarrhea resolution [19,20,23,25,27].

Most of the patients were only mildly dehydrated, had a non-bacterial cause of their diarrhea, and had diarrhea for fewer than 3 d prior to enrolment in the studies (Table 1). Most (11/13) studies reported that patients received oral rehydration therapy in addition to the study medication [17,19–21,23–27,31]. Only four studies specifically mentioned that some of their patients received intravenous fluid rehydration in addition to oral rehydration therapy [17–19,27]; two studies did not report the hydration method used [16,22]. Early refeeding was reported in four studies [23,24,26,31]; restriction to only oral rehydration therapy in the first 24 h was reported in two studies [20,25]; and six studies did not report on feeding practices [16–19,22,27].

### Methodological Quality of Trials

Generation of the allocation sequence was reported in only six out of 13 studies (Table 2) [17,19,21,25,26,31]. Allocation concealment was reported in seven studies [17,19–21,25,26,31]. Although nine trials were double-blind [16,17,19–21,25–27,31], four were open [18,22–24]. Most (11/13) trials reported inclusion of more than 90% of all randomized participants in their analysis [17–26,31]. Only six studies met all four indicators of methodological quality that we used [17,19,21,25,26,31], and of those, only four provided outcome data that could be combined [17,19,21,31].

**Table 2:**
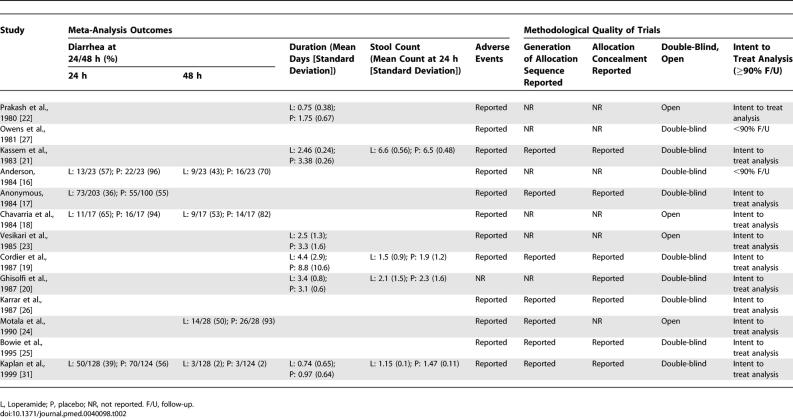
Outcomes of Studies Included in the Meta-Analysis of Use of Loperamide for Acute Diarrhea and Methodological Quality of Trials

### Quantitative Data Synthesis

#### Diarrhea continuing at 24 and 48 h.

**Figure 2:**
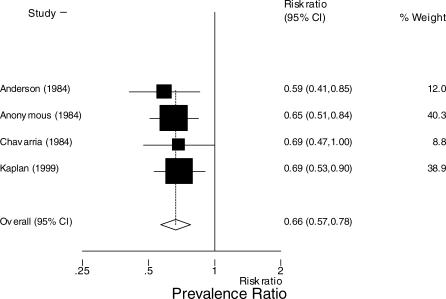
Meta-Analysis of Prevalence of Diarrhea at 24 h among Those on Loperamide Compared with Controls The *x*-axis uses the log scale. Random-effects prevalence ratio 0.66 (95% CI: 0.57 to 0.77). Test for heterogeneity, *p* = 0.914.

**Figure 3:**
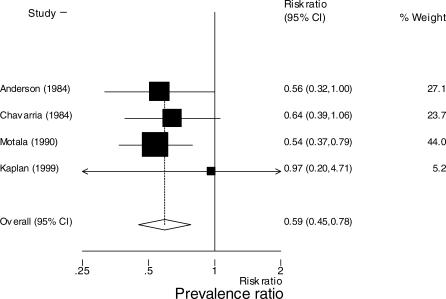
Meta-Analysis of Prevalence of Diarrhea at 48 h among Those on Loperamide Compared with Controls The *x*-axis uses the log scale. Random-effects prevalence ratio 0.58 (95% CI: 0.45 to 0.76). Test for heterogeneity, *p* = 0.864.

Data on diarrhea continuing at 24 or 48 h were available in five studies [16–18,24,31]. In the four studies reporting diarrhea at 24 h, the prevalence of diarrhea among patients on loperamide was less than among patients on placebo; prevalence ratio 0.66 (95% CI: 0.57 to 0.78) (Figure 2) [16–18,31]. When the meta-analysis was restricted to the three studies that used the same definition of diarrhea resolution, last unformed stool, the prevalence of diarrhea among patients on loperamide was less than among patients on placebo: prevalence ratio 0.66 (95% CI: 0.56 to 0.77) [16,17,31]. In the four studies reporting diarrhea at 48 h among patients on loperamide, the prevalence of diarrhea was less than among patients on placebo: prevalence ratio 0.59 (95% CI: 0.45 to 0.78) (Figure 3) [16,18,24,31].

#### Mean reduction in diarrhea duration.

**Figure 4:**
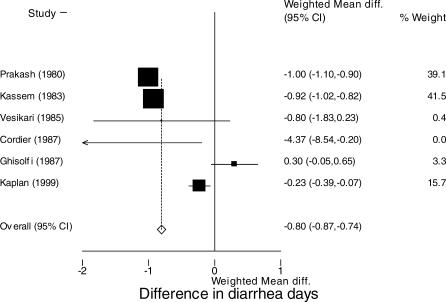
Diarrhea Duration Reduction of diarrhea duration (days) in six included studies that reported mean reduction and variance using the fixed-effects method: 0.8-d reduction in diarrhea (95% CI: 0.74 to 0.87 d). Box size is proportional to the inverse magnitude of the study variance. Random-effects reduction in diarrhea duration: 0.56 d (95% CI: 0.16 to 0.95 d). Test for heterogeneity, *p* < 0.001.

Data on diarrhea duration were available for six studies [19–23,31]. Among those on loperamide, compared with those assigned to placebo, mean diarrhea duration was 0.8 d shorter, (95% CI: 0.7 to 0.9 d) (Figure 4) [19–23,31]. Similar values for mean reduction in diarrhea duration were estimated when restricted to the five studies with a loperamide dose of ≤0.25 mg/kg/d (mean reduction in diarrhea duration 0.7 d, 95% CI: 0.6 to 0.8 d) [19–21,23,31].

#### Diarrhea frequency.

**Figure 5:**
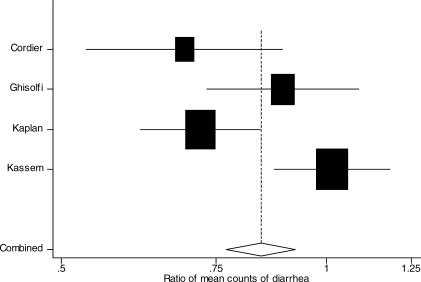
Meta-Analysis of Ratio of Mean Counts of Diarrheal stools at 24 h among Those on Loperamide Compared with Controls The *x*-axis uses the log scale. Fixed-effects ratio of count of stools at 24 h: 0.84 (95% CI: 0.77 to 0.92). Random-effects ratio of count of stools at 24 h: 0.83 (95% CI: 0.69 to 1.00). Test for heterogeneity, *p* < 0.001

Data on diarrhea counts in the first 24 h were available for four studies. Of these four studies, data on diarrhea counts were available in 8-h intervals in three studies [19,20,31] and in 24-h intervals in one study [21]. The count of stools in the first 24 h was lower among patients on loperamide compared with patients on placebo: count ratio 0.84 (95% CI: 0.77 to 0.92) (Figure 5) [19–21,31].

#### Adverse events.

Data on adverse events were available from 12 studies [16–19,21–27,31]. Adverse events were reported in 94 out of 927 patients (10.1%, 95% CI: 8.3% to 12.3%) allocated to loperamide and in 16 out of 764 patients (2.1%, 95% CI: 1.2% to 3.4%) allocated to placebo (risk difference 8.6% [95% CI: 6.4% to 10.9%]) (Table 3). Serious adverse events, defined as ileus, lethargy, or death, occurred only among children younger than 3 y of age [23,24,26]. We could not estimate age-specific prevalence of adverse events since counts of adverse events by age of child were not reported. Serious adverse events were reported in eight out of 927 children allocated to loperamide (0.9%, 95% CI: 0.4% to 1.7%) but were not reported in any of the 764 children allocated to placebo (0%, 95% CI: 0% to 0.5%): risk difference 0.8% (95% CI: −0.1% to 1.8%). Lethargy was reported in four children younger than 3 y given 0.1 mg/kg/d of loperamide [23]. The other serious adverse events were in children younger than 3 y given 0.4–0.8 mg/kg/d of loperamide: ileus in a 4-mo-old infant [24], lethargy in two children [26], and death from complications from Salmonella typhi bacteremia in one child [26].

**Table 3:**
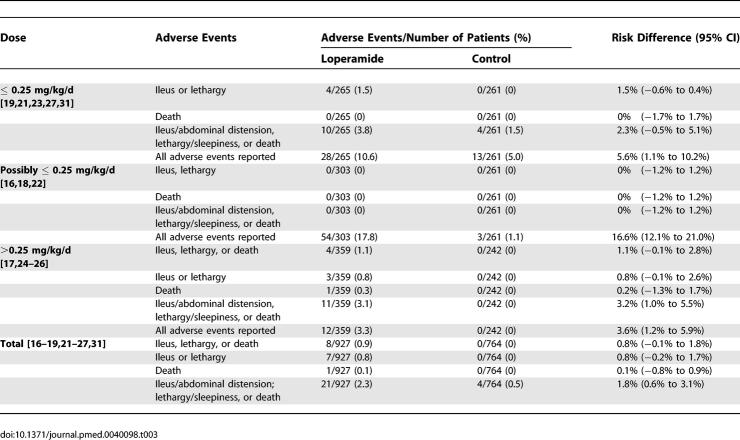
Adverse Events: Prevalence of Adverse Events in Patients Treated with Loperamide or Placebo

When the definition of serious adverse events was expanded to include abdominal distension and sleepiness, adverse events were recorded in 21 out of 927 children (2.3%, 95% CI: 1.4% to 3.4%) allocated to loperamide, and in four out of 764 children (0.5%, 95% CI: 0.1% to 1.3%) allocated to placebo (risk difference 1.8%, 95% CI: 0.6% to 3.1%). Sleepiness was reported in seven patients, six of whom were allocated to loperamide (0.25–0.8 mg/kg/day), with one child allocated to placebo [24,31]. Of the children with sleepiness recorded, four children allocated to loperamide 0.8 mg/kg/d were younger than 3 mo of age [24]; the ages of the other children with sleepiness were not reported. Abdominal distension was reported in ten children under the age of 3 y, seven of whom were allocated to loperamide (0.24–0.8 mg/kg/day), with three allocated to placebo [17,19,21,26].

#### Sensitivity analysis.

We did not find any important changes in our estimates when we restricted our analyses to studies with loperamide at a dose of ≤0.25 mg/kg/d, studies on outpatient populations, studies that fulfilled all four indicators of methodological quality, studies that defined diarrhea resolution, or studies with fewer than 25% of diarrhea cases attributed to a bacterial pathogen (Table 3).

In addition, we did not find any changes in our overall estimates when using the random-effects method (Table 4). The smallest *p*-value for tests of homogeneity was 0.6 when examining studies reporting diarrhea continuing at 24 or 48 h. Results from studies reporting differences in diarrhea duration (expressed as a continuous variable) and diarrhea frequency were more disparate; the *p*-values for tests of homogeneity were <0.01. All inferences continued to be statistically significant with the random-effects method except in sub-analyses (Table 3). Using the random-effects method, the sub-analyses of the mean differences in diarrhea duration were no longer statistically significant in meta-analyses of studies of loperamide doses of ≤0.25 mg/kg/d, in meta-analyses of studies that met all four indicators of methodological quality, or in meta-analyses of studies with <25% diarrhea cases attributed to a bacterial pathogen (Table 3). Using the random-effects method, the sub-analyses of the mean differences of diarrhea count at 24 h were no longer statistically significant in meta-analyses of studies that met all four indicators of methodological quality (Table 4). None of the factors examined in the sub-analyses could account for study heterogeneity in the studies of diarrhea duration or in the studies of diarrhea frequency; the *p*-values of tests of homogeneity of these subgroups remained <0.01. We were unable to test whether a consistent definition of diarrhea resolution could account for study heterogeneity since no two of the studies of diarrhea duration or diarrhea frequency defined diarrhea resolution in the same way.

**Table 4:**
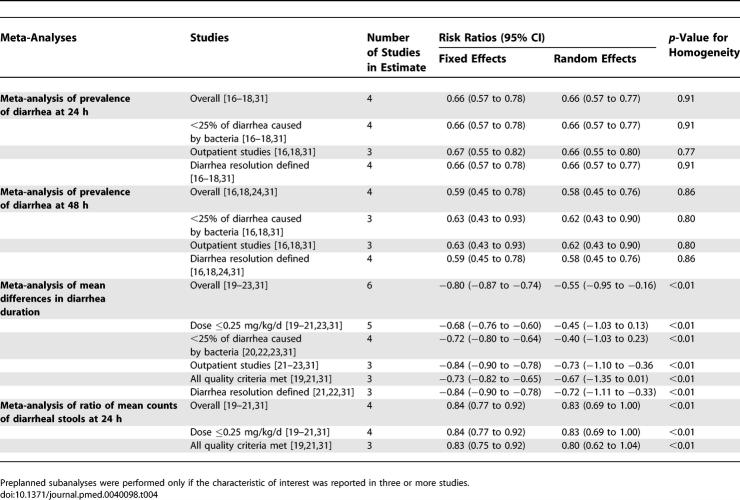
Meta-Analysis Results among Those on Loperamide Compared with Controls

## Discussion

Loperamide appears to decrease diarrhea duration and frequency in children when used as an adjunct to oral or intravenous rehydration. Compared with patients given placebo, patients who were randomized to loperamide were 34% less likely to have had diarrhea 24 after initiation of therapy and 41% less likely to have had diarrhea 48 h after initiation of therapy, had 0.8 fewer days of diarrhea, and had a 16% reduction in diarrheal stools within the first 24 h of treatment. Serious adverse events of death, ileus, or lethargy were reported only in children younger than 3 y of age.

Our review should be evaluated with the following limitations in mind. First, although our review drew from an extensive search and was not limited by language, conference proceedings were not searched. Therefore, it is possible that additional information from conference proceedings that did not lead to a separate publication and that were not noted by researchers in the field was missed.

Second, our meta-analysis was limited by a lack of consistency in outcome measures, and this restricted our ability to combine results across many trials. Although we report the results of 13 studies, not all studies presented outcomes that could be summarized. Stool volume could not be summarized because only one study reported stool volume with standard deviations. In addition, for many outcomes, no results were reported. We were unable to examine whether patients received intravenous fluid administration or hospitalization because these outcomes were so rarely reported. Studies did not report whether loperamide prevented the progression from acute to persistent diarrhea.

Third, we found evidence of heterogeneity for some outcomes. We could not account for the observed heterogeneity in several subgroup analyses. Despite this heterogeneity, both fixed-effects and random-effects summaries were consistent with beneficial effects of loperamide in the overall estimates; the heterogeneity suggests the size of the benefit varies by some other factor that we were not able to identify.

Fourth, it is unclear how representative the children recruited in the studies were compared with all children worldwide with diarrhea. Although many studies included children with mild dehydration, several studies did not report hydration status [16,18,22,31] or specifically excluded children with moderate [31] or severe dehydration [22,24,25,31] (Table 1). No study reported nutritional status, and several trials specifically excluded patients who were malnourished [21,22,24,25]. While most studies included patients with diarrhea from bacterial sources, several studies did not report diarrhea etiology or specifically excluded patients who had bloody diarrhea [24,25,31]. In addition, several studies specifically excluded patients who were systemically ill [21,22,24,25,31] or required antibiotics [21,24,31].

Finally, although serious adverse events were reported only in children younger than 3 y of age, age-specific prevalence of adverse events could not be calculated since studies reported neither the count of adverse events by age of child nor the count of total number of children in each age category. Our reported prevalence of serious adverse events is an underestimate for children younger than 3 y and an overestimate for children 3 y and older. In addition, randomized controlled studies may not be the best way to determine the incidence of rare adverse events. We provided both prevalence of serious adverse events (ileus, lethargy, and death) and prevalence of an expanded version of serious adverse events (including sleepiness and abdominal distension) because the distinctions between sleepiness and lethargy, and between abdominal distension and ileus, were not defined in the studies. Before using loperamide, health-care providers and parents should consider the potential benefit of whether decreasing diarrhea duration by 1 d is worth the potential risk of adverse events. Children who were malnourished, had bloody diarrhea, were systemically ill, or were moderately/severely dehydrated were often excluded from these trials, so loperamide should probably not be used in that population. For children younger than 3 y, the risk of serious adverse events probably outweighs the benefit of a potential 1-d reduction in diarrhea duration. In addition, since loperamide appears efficacious in doses as low as 0.1–0.25 mg/kg/d, the lowest efficacious dose should be used to try to minimize the risk of overdosing loperamide.

The results of our systematic review and meta-analysis are consistent with a previous review on the efficacy and safety of loperamide in adults [32]. In their 1990 review of the efficacy and safety of loperamide for acute diarrhea in adults, Ericsson et al. concluded that loperamide is effective and safe for the treatment of diarrhea [32]. The authors reviewed five studies of loperamide among persons of 8 y and older and included studies that compared loperamide to another drug (such as diphenoxylate or bismuth subsalicylate) [32]. To our knowledge, our study is the first systematic review and meta-analysis that focuses on the efficacy and safety of loperamide in children.

Future studies of diarrhea might benefit from clear definitions of minimum diarrhea severity for inclusion in a trial, definitions of diarrhea resolution, and outcomes with means and standard deviations which could be combined, such as diarrhea duration, count of diarrheal stools at 24 h, and number of patients with diarrhea resolution at 24 h. If these definitions and outcomes were used consistently across studies on acute diarrhea, studies would be more amenable to being summarized with meta-analysis techniques. Logrank tests (a comparison of the survival curves showing how quickly diarrhea resolved in the loperamide and control groups) [13] may be the most appropriate test in some situations; however, including an outcome that could be readily combined in meta-analysis may be helpful. In addition, future studies of children might benefit from presenting results and adverse event data by year of age and treatment arm, as age may modify a child's response to treatment and risk of adverse events.

Oral rehydration therapy and early refeeding should remain the focus of management of diarrhea. Loperamide may be considered as an adjunct to oral rehydration therapy and early refeeding. Since diarrhea is usually a self-limited disease in industrialized societies, physicians and families should weigh the possibility of adverse events against a modest improvement in diarrhea. In children who are younger than 3 y, malnourished, moderately or severely dehydrated, systemically ill, or have bloody diarrhea, adverse events outweigh benefits even at doses ≤0.25 mg/kg/d. In children who are older than 3 y with no/minimal dehydration, loperamide may be a useful adjunct to oral rehydration and early refeeding.

## Supporting Information

Text S1 QUOROM Checklist (33 KB DOC)

## Abbreviation

CI: confidence interval
