# Supplementary material for: Loperamide Therapy for Acute Diarrhea in Children: Systematic Review and Meta-Analysis
Source: PLoS Med. 2007 Mar 27;4(3):e98. doi: 10.1371/journal.pmed.0040098 (PMC1831735; doi:10.1371/journal.pmed.0040098)
Supplement: Text S1 — (33 KB DOC) [file pmed.0040098.sd001.doc]

| **QUOROM Statement checklist Loperamide Therapy for Acute Diarrhea in Children: Systematic Review and Meta-analysis** | | | | |
| --- | --- | --- | --- | --- |
| **Heading** | **Subheading** | **Descriptor** | **Reported? (Y/N)** | **Heading: Subheading** |
| **Title** |  | Identify the report as a systematic review | Y | Title |
| **Abstract** |  | Use a structured format | Y | Abstract |
|  | Objectives | The clinical question explicitly | Y | Abstract: Methods and Findings |
|  | Data sources | The databases (ie, list) and other information sources | Y | Abstract: Methods and Findings |
|  | Review methods | The selection criteria (ie, population, intervention, outcome, and study design); methods for validity assessment, data abstraction, and study characteristics, and quantitative data synthesis in sufficient detail to permit replication | Y | Abstract: Methods and Findings |
|  | Results | Characteristics of the RCTs included and excluded; qualitative and quantitative findings (ie, point estimates and confidence intervals); and subgroup analyses | Y | Abstract: Methods and Findings |
|  | Conclusion | The main results | Y | Abstract: Methods and Findings |
|  |  |  |  |  |
|  |  | **Describe** |  |  |
| **Introduction** |  | The explicit clinical problem, biological rationale for the intervention, and rationale for review | Y | Introduction |
| **Methods** | Searching | The information sources, in detail (eg, databases, registers, personal files, expert informants, agencies, hand-searching), and any restrictions (years considered, publication status, language of publication) | Y | Materials and Methods: Data Sources |
|  | Selection | The inclusion and exclusion criteria (defining population, intervention, principal outcomes, and study design | Y | Materials and Methods: Study Selection, Outcome Measures |
|  | Validity assessment | The criteria and process used (eg, masked conditions, quality assessment, and their findings) | Y | Materials and Methods: Methodological quality of studies |
|  | Data abstraction | The process or processes used (eg, completed independently, in duplicate) | Y | Materials and Methods: Data abstraction |
|  | Study characteristics | The type of study design, participants' characteristics, details of intervention, outcome definitions, and how clinical heterogeneity was assessed | Y | Materials and Methods: Study characteristics |
|  | Quantitative data synthesis | The principal measures of effect (eg, relative risk), method of combining results (statistical testing and confidence intervals), handling of missing data; how statistical heterogeneity was assessed; a rationale for any a-priori sensitivity and subgroup analyses; and any assessment of publication bias | Y | Materials and Methods: Statistical Methods |
| **Results** | Trial flow | Provide a meta-analysis profile summarising trial flow (see figure) | Y | Results: Study Selection; Figure 1 |
|  | Study characteristics | Present descriptive data for each trial (eg, age, sample size, intervention, dose, duration, follow-up period) | Y | Results: Study Characteristics; Table 1 |
|  | Quantative data synthesis | Report agreement on the selection and validity assessment; present simple summary results (for each treatment group in each trial, for each primary outcome); present data needed to calculate effect sizes and confidence intervals in intention-to-treat analyses (eg 2X2 tables of counts, means and SDs, proportions) | Y | Results: Quantitative data synthesis; Tables 2, 3, 4 |
| **Discussion** |  | Summarise key findings; discuss clinical inferences based on internal and external validity; interpret the results in light of the totality of available evidence; describe potential biases in the review process (eg, publication bias); and suggest a future research agenda | Y | Discussion |
